# Expansion by whole genome duplication and evolution of the *sox* gene family in teleost fish

**DOI:** 10.1371/journal.pone.0180936

**Published:** 2017-07-24

**Authors:** Emilien Voldoire, Frédéric Brunet, Magali Naville, Jean-Nicolas Volff, Delphine Galiana

**Affiliations:** Institut de Génomique Fonctionnelle de Lyon, Université de Lyon, Université Lyon1, CNRS, Ecole Normale Supérieure de Lyon, Lyon, France; Universite de Rouen, FRANCE

## Abstract

It is now recognized that several rounds of whole genome duplication (WGD) have occurred during the evolution of vertebrates, but the link between WGDs and phenotypic diversification remains unsolved. We have investigated in this study the impact of the teleost-specific WGD on the evolution of the *sox* gene family in teleostean fishes. The *sox* gene family, which encodes for transcription factors, has essential role in morphology, physiology and behavior of vertebrates and teleosts, the current largest group of vertebrates. We have first redrawn the evolution of all *sox* genes identified in eleven teleost genomes using a comparative genomic approach including phylogenetic and synteny analyses. We noticed, compared to tetrapods, an important expansion of the *sox* family: 58% (11/19) of *sox* genes are duplicated in teleost genomes. Furthermore, all duplicated *sox* genes, except *sox17* paralogs, are derived from the teleost-specific WGD. Then, focusing on five *sox* genes, analyzing the evolution of coding and non-coding sequences, as well as the expression patterns in fish embryos and adult tissues, we demonstrated that these paralogs followed lineage-specific evolutionary trajectories in teleost genomes. This work, based on whole genome data from multiple teleostean species, supports the contribution of WGDs to the expansion of gene families, as well as to the emergence of genomic differences between lineages that might promote genetic and phenotypic diversity in teleosts.

## Introduction

The origin and evolution of biodiversity remain fundamental questions in biology [1]. At the genetic level, the importance of gene duplication in innovation and diversification has been recognized for several decades [2–4]. Especially, the duplication of all genes through whole-genome duplications (WGDs) corresponding to polyploidization events could provide a particularly remarkable opportunity for the emergence of evolutionary novelties and functional diversification [5, 6]. Even if paleopolyploidy events are rare [6], several rounds of ancient WGD have been inferred in vertebrates, including two WGDs at the base of the vertebrate lineage [4, 7]. Moreover, it is now well established that an additional WGD, also known as the teleost-specific WGD, occurred in the lineage leading to teleostean fishes about 250 mya [8–10]. More recent and lineage-specific WGD events have been also inferred in various vertebrate lineages, such as in salmonids and Xenopus [11].

Polyploidization that leads to the doubling of the whole genome content is evolutionarily unstable and followed by a re-diploidization process (whereby tetraploid species become diploid again) that progressively reduces the gene number [12]. Re-diploidization involves many genomic rearrangements such as inversions and/or deletions [13]. In addition, as the two gene copies produced by WGD are originally redundant, one copy can accumulate degenerative mutations and be eliminated by a process called non-functionalization [14]. However, genomic studies in various taxa have shown that, after an event of WGD, 10 to 30% of duplicated genes are preserved after re-diploidization [15–17]. The two main and non-exclusive models explaining duplicated gene retention are sub-functionalization, in which the ancestral properties (function and/or expression) are re-distributed between the two copies, and neo-functionalization, when one of the two copies acquires a new expression regulation or function [18]. It has been proposed that divergent resolution of gene duplicates, *id est* lineage-specific gene deletion and/or divergent sub/neo-functionalization events, might have occurred among lineages that diverged after the WGD. Consequently, WGDs followed by divergent resolution might have played an important role in the emergence of structural and functional genomic differences, and may contribute to genetic and phenotypic diversity, and consequently to speciation [6, 14, 19, 20]. In plants, there is a strong correlation between increased rates of speciation and the occurrence of WGDs [15, 21–24]. In animals, the correlation between WGDs, divergent evolution of paralogs and species enrichment, mostly supported by bioinformatics analyses, is less clear. *Xenopus laevis*, which underwent a WGD event approximately 40 million years ago and maintained more than 30% of its genes in duplicate, seems to bring evidence supporting this hypothesis [25–27]. However, we still lack clear experimental examples of these causal relations.

Teleostean fish, which represent more than half of the extant vertebrate species, constitute a relevant group to search for concrete molecular proofs that could link WGD to biodiversity in vertebrates. Indeed, recent studies have demonstrated the contribution of the teleost-specific WGD to gene family expansion in teleosts [28–30]. Moreover, whole-genome sequence data from multiple teleostean species are now available as well as reliable phylogenetic frameworks. These new data provide us with the opportunity to analyze the fate of WGD-derived duplicated genes and gene families over long evolutionary periods in teleosts.

In this study, we analyzed the impact of the teleost-specific WGD on the evolution of the *sox* gene family, which encodes for transcription factors. The *SRY*-related HMG box (or *sox*) gene family, a metazoan-specific gene family, has expanded over time in a lineage-specific manner through duplication events. In mammals, the family is composed of twenty members [31] sorted into eight groups (A, B1, B2, C, D, E, F, and H). This ancient family of transcription factors is implicated in fundamental functions during embryogenesis, but also in adult homeostasis in mice [32]. The mammal-specific *Sry* gene (the unique representative of the group A), which was the first *sox* member identified approximately 25 years ago [33, 34], is an excellent example of the importance of the *sox* family: *Sry* initiates the male sex-determination gene cascade during gonads development in mammals [35]. The seven other groups mentioned above are present in all bilaterians, generally represented by a single *sox* gene per group in invertebrates [36]. In contrast, vertebrate genomes contain for all groups, except group H represented only by one gene, two to four *sox* genes, as a probable consequence of the two rounds of WGD that occurred at the base of vertebrates [6, 37]. In addition, many studies have shown that several vertebrate *sox* genes are duplicated in different teleostean genomes, and especially in the zebrafish [29, 37–46]. These studies also suggest that most of the *sox* teleost-specific WGD duplicates have been preserved in genomes thanks to sub-functionalization, often in a lineage-specific manner [39, 40, 42]. However, the impact of the teleost-specific WGD on the whole *sox* family remains unknown.

In this context, the aim of this work was first to estimate the extend of *sox* gene expansion in the teleost lineage, *id est* to draw, by comparative genomics, the landscape of all *sox* genes in all available teleost genomes, and to determine the contribution of the teleost-specific WGD to the evolution of the *sox* gene family. Second, we investigated the evolution of some *sox* duplicates in teleost genomes by analyzing their genomic environment and their expression pattern during development and adulthood.

## Material and methods

### Fish

Platyfish (*Xiphophorus maculatus*, population Usumacinta), zebrafish (*Danio rerio*, strain AB/TU) and medaka (*Oryzias latipes*, strain Hd-rR) were kept under standard conditions at the PRECI aquarium facility of the SFR Biosciences Gerland-Lyon Sud (Lyon, France). Fish embryos were raised to the required stages of development in E3 embryo media [47] at 26°C for medaka and 28.5°C for zebrafish. Medaka and zebrafish embryos were staged according to Iwamatsu [48] and Kimmel [49], respectively. R. Guyot (IGFL, ENS de Lyon, France) and M. Teixeira (PBES, ENS de lyon, France) provided mouse samples from strain OF1. Experimental procedures have been performed following protocols in accordance with regulations from the French Ministry of Agriculture and the European Union (agreement number A693870602).

### Sacrifice of fish for sampling

The adult fish (medaka, zebrafish and platyfish) are incubated in their usual water containing 0.4 g/L of tricaine methanesulfonate (MS-22) for 10 minutes. Death is observed by cessation of opercular movements.

### Sequence retrieval and alignment

Nucleotide sequences of *sox* genes were obtained using TBlastN [50] searches against GenBank, Trace Archives Nucleotide from the National Center for Biotechnology Information (www.ncbi.nlm.nih.gov), Harvard Expressed Sequence Tags bank (http://compbio.dfci.harvard.edu/tgi/) and Ensembl genome assemblies (www.ensembl.org) of the mouse (*Mus musculus*), coelacanth (*Latimeria chalumnae*), spotted gar (*Lepisosteus oculatus*), and the nine teleosts zebrafish (*Danio rerio*), cave fish (*Astyanax mexicanus*), cod (*Gadus morhua*), tilapia (*Oreochromis niloticus*), medaka (*Oryzias latipes*), platyfish (*Xiphophorus maculatus*), Tetraodon (*Tetraodon nigroviridis*), fugu (*Takifugu rubripes*) and stickleback (*Gasterosteus aculeatus*). *Sox* sequences from the salmon (*Salmo salar*), the rainbow trout (*Oncorhynchus mykiss*) and the elephant shark (*Callorhinchus milii*) were obtained at http://web.uvic.ca/grasp/gils/, www.sigenae.org, and http://esharkgenome.imcb.a-star.edu.sg/, respectively. *Sox* genes along with their accession numbers and/or coordinates are available in S1 Table. Nucleotide sequences were loaded into MEGA [51], translated into proteins and aligned using ClustalW [52, 53]. Sequence alignments were further refined manually using Seaview (version 3.2; [54]).

### Phylogenetic reconstructions

Maximum likelihood phylogenies were computed based on protein sequences with PhyML implemented in Seaview (version 3.2; [54]). LG model of protein evolution was used and topology optimization was carried out using best of NNI and SPR option. Default aLRT (SH-like) was used for branch support [55]. For *sox19*, sequence alignments were too short to perform relevant phylogenetic analyses.

### Synteny analyses

Macrosynteny analyses were performed on four sequenced teleosts: zebrafish *D*. *rerio*, medaka *O*. *latipes*, stickleback *G*. *aculeatus* and Tetraodon *T*. *nigroviridis* using their last release of genome assemblies in Ensembl. For each genome, a rose window representation was generated [9, 16, 29]. When genes were not assigned to chromosomes or linkage group, they were not included in the rose window representation.

### Evolutionary rate computations

Alternative models with different branch-specific dN/dS ratio were compared using the codeml program of PAML [56]. The data set includes two mammals (*Mus musculus* and *Homo sapiens*), the chicken *Gallus gallus* and five species of teleosts (zebrafish, medaka, platyfish, tilapia and fugu). When available, sequences from the frog *Xenopus tropicalis*, the two non-teleost fishes spotted gar and coelacanth, and the six teleost fishes salmon, Tetraodon, cod, stickleback, orange-spotted grouper (*Epinephelus coioides*) and african sharp tooth (*Clarias gariepinus*) were included into the analyses. Alignments based on protein sequences were produced with ClustalW implemented in MEGA [51] and computations were performed on the alignments after removal of positions presenting at least one gap. Two analyses were performed: 1) the model A, in which a single dN/dS ratio (also named ω in S4 Fig) is estimated for all branches, was compared with the model B, in which a higher dN/dS ratio is estimated for fish paralogs a and b compared to vertebrate orthologs; 2) the model B was compared with the model C, in which three dN/dS ratios are estimated: one dN/dS ratio for fish paralogs a, one dN/dS ratio for fish paralogs b and one dN/dS ratio for vertebrate orthologs. *Sox19* was not included as sequences were too short to perform relevant analyses.

### Search for conserved non-coding elements in *sox* gene environment

Two complementary methods were applied to look for conserved non-coding elements (CNEs) in the vicinity of fish *sox* genes. First, non-coding elements conserved at a large scale in vertebrates were identified in the 46-species multiple alignments obtained from the UCSC (University of California, Santa Cruz) using a homemade algorithm (written by H. Roest Crollius, ENS Paris, and adapted by M. Naville). Basically, the program scans the alignment and looks for conserved regions of a minimal length (10 bp) and identity (90% in the 10 bp-seed region, further extended by accepting up to 3 non-conserved columns on each side). The program allows substitutions to occur under a threshold of 12% in each column of the alignment. It was applied apart from any exon and human masked sequences (repeatmasker or tandem repeats) to 1Mb regions upstream and downstream of *SOX* genes in human. Elements found in at least one fish were retained. Second, ray-finned fish-specific CNEs were searched in 800kb regions centered on the different *sox* genes using Blastn and the spotted gar region, cleaned from any exonic and UTR elements, as query against 6 other fish *sox* regions (zebrafish, medaka, tilapia, platyfish, Tetraodon and fugu), with the following parameters: gap opening 2, gap extension 2, mismatch penalty -1, match reward 1. Only Blast hits longer than 50 bp and at least 60% identical to the spotted gar sequence were retained.

### RT-qPCR expression analyses

Total RNA was extracted from separated male and female tissues (spleen, liver, ovaries/testes, muscle, eyes, brain) and from whole embryos using the TRIReagent (Molecular Research Center, Inc.). After DNase treatment, four micrograms of total RNA were reverse-transcribed with the RevertAid First Strand Synthesis kit (Fermentas) using random hexamer primers. Real-time PCR was performed on 2 μL of cDNA starting from a dilution factor of 15 using IQTM Custom SYBR^®^ Green Supermix (Bio-rad). PCR amplification was monitored with a CFX96 Realtime System (Bio-rad). Cycling conditions were 95°C for 2 min, 40 cycles at 95°C for 10 seconds and 59°C for 30 seconds. Three replicates were performed per sample. Quantification cycle (Cq; standard name for Ct or Cp value) values were recorded with the software Bio-rad CFX manager version 2. Cq values ≥ 34 were considered beyond the limit of detection (a Cq value of 33 represents a single molecule template detection). The 2 genes *bActin2* and *rpl7* (*ribosomal protein l7*) were found to be the most stable reference genes respectively for embryos and adults (data not shown), and were used to normalize the data. Data were analyzed by the ΔΔCT method [57].

## Results

### The *sox* gene landscape in teleostean genomes

**Fig 1:**
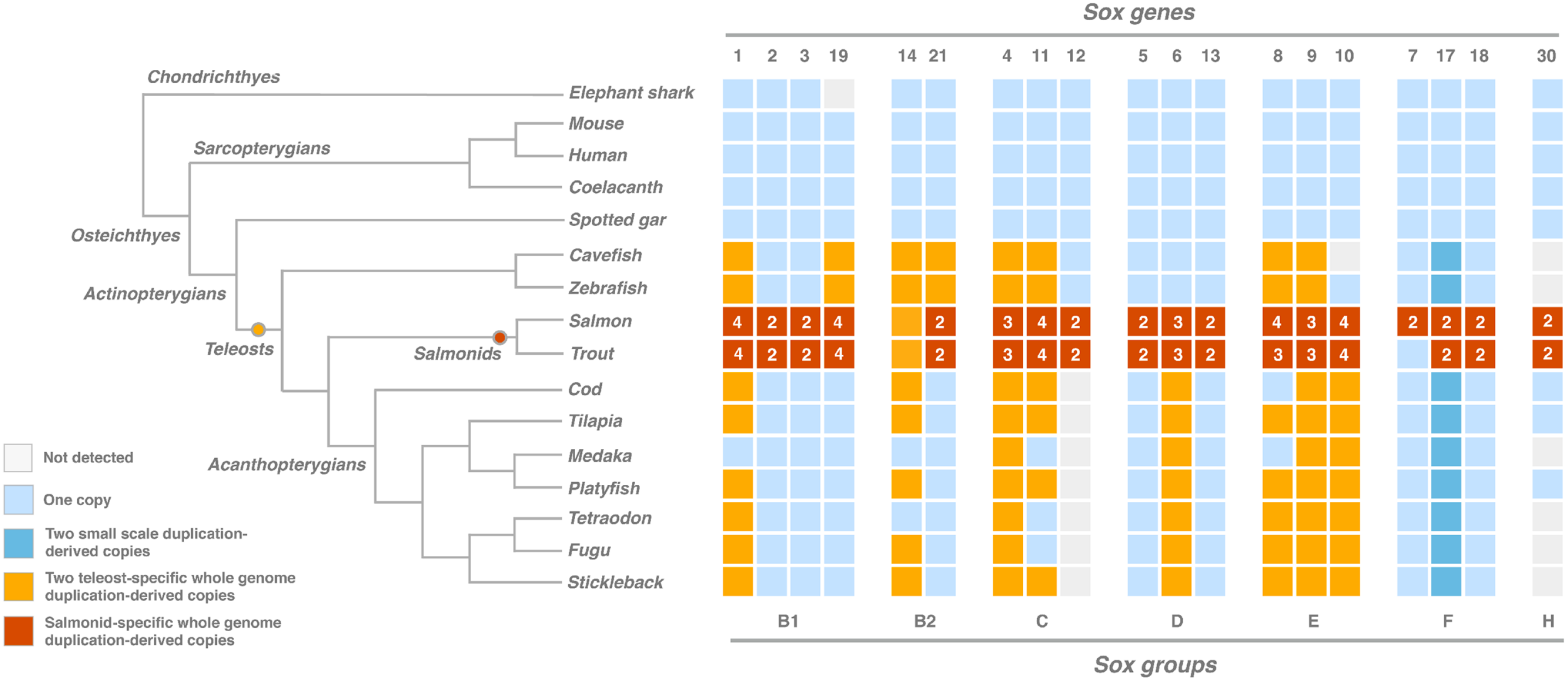
Landscape of *sox* genes in representative vertebrate genomes. The *sox* genes (top) are divided into the 7 groups: B1, B2, C, D, E, F and H. Phylogenetic relationships of the different analyzed vertebrate species are indicated on the left. The orange and red circles on the phylogeny represent the teleost-specific WGD and the salmonid-specific WGD, respectively. Light blue squares indicate gene singletons. Orange and dark blue squares indicate duplicates produced either by the teleost-specific WGD or by small-scale duplications (SSDs) respectively. Red squares correspond to genes detected in multiple copies (two, three or four as indicated by the number in the square) in salmonids. White squares are used when no copy was detected. The mammal-specific *SoxA* group is not represented on the figure.

*Sox* genes were searched genome-wide (see Materials and methods section) in the eleven most complete teleost genomes (Fig 1). The same analyses were performed in five non-teleost species (elephant shark, mouse, human, coelacanth and spotted gar) in order to gain insight into the evolutionary dynamics of this gene family in vertebrates. We used the sequences of human *SOX* genes (except that of *SRY*, which is eutherian mammal-specific [58]) as queries. The obtained landscape is presented in Fig 1. The spotted gar, which is the closest fish related to teleosts with sequenced genome that did not experience the teleost-specific WGD [59], contains nineteen *sox* genes in its genome. These nineteen *sox* genes are also present as singletons in the three other Sarcopterygii analyzed (coelacanth, mouse and human). Of note, the elephant shark, a cartilaginous fish, seems to have lost *sox19*. Concerning teleosts, the analysis indicates enrichment in *sox* genes compared to other vertebrates. Indeed, not considering salmonids which are particular (see below), 57.8% (11/19) of *sox* genes are duplicated in at least one teleost species. In more details, 27.3% (3/11) are duplicated in all the species studied, whereas 72.7% (8/11) are duplicated only in particular lineages or species. Furthermore, on the nineteen vertebrate *sox* genes used as query, some seem to be absent in particular teleostean species or lineages, as no corresponding sequences have been identified for the vertebrate *sox10* gene in the cavefish genome, for *sox12* gene in the acanthopterygian genomes (cod, tilapia, medaka, platy, tetraodon, fugu and stickleback), and for *sox30* gene in six teleost genomes (cavefish, zebrafish, medaka, Tetraodon, fugu and stickleback). Finally, salmon and trout possess more *sox* genes (51 and 49, respectively) than the other teleostean species (from 22 to 27) presented in this study, probably due to the specific WGD that occurred in the salmonidae lineage about 100 mya [60].

### Origin of *sox* gene duplicates in teleosts

**Fig 2:**
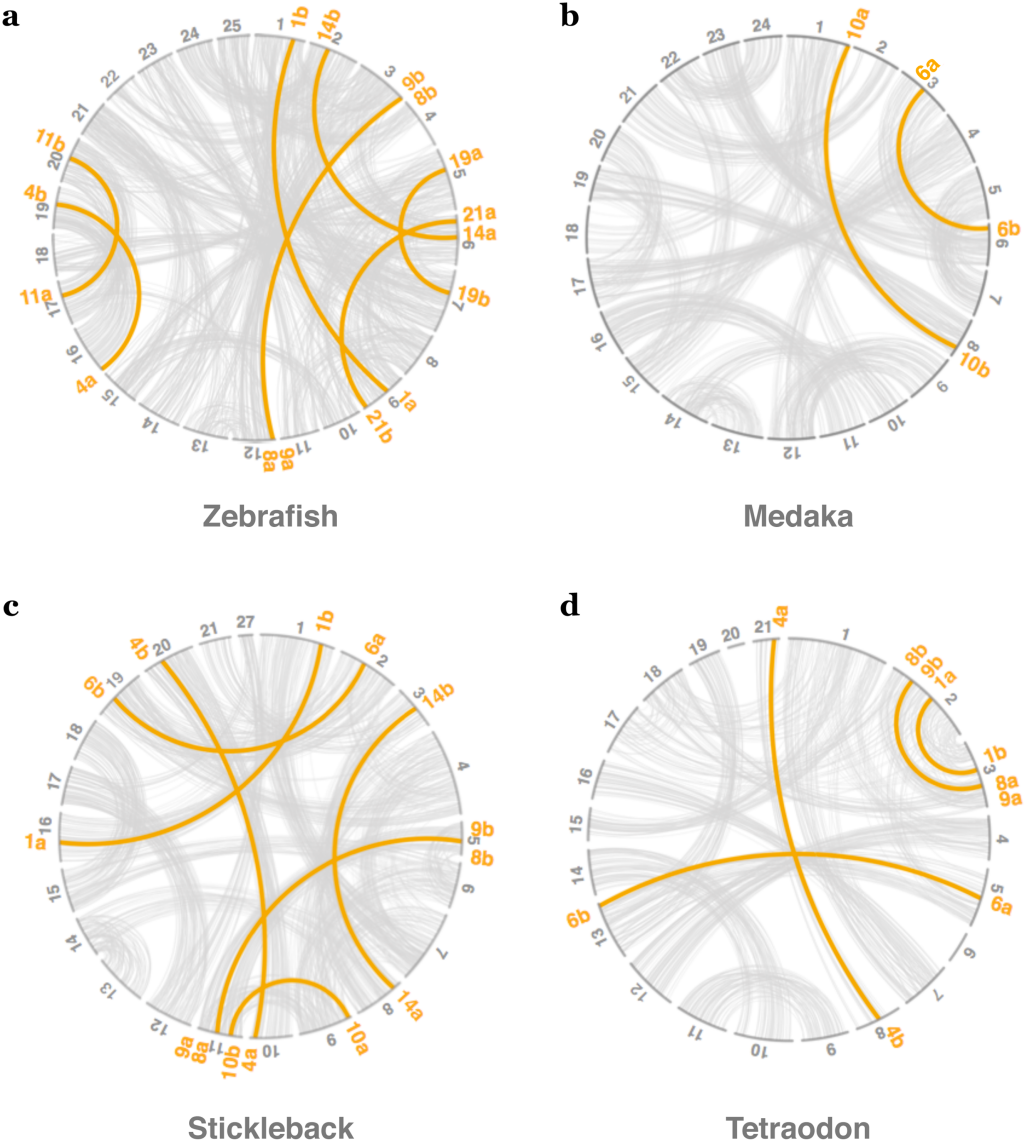
Macrosynteny analyses of duplicated *sox* genes in teleosts. (a-d) All *sox* duplicates annotated in the four zebrafish, medaka, stickleback and Tetraodon genomes were studied in the macrosynteny analysis using the last release of genome assemblies in Ensembl. Grey lines connect paralog genes on the different chromosomes or linkage group in the genomes. Orange lines connect paralog *sox* genes on different chromosomes.

**Fig 3:**
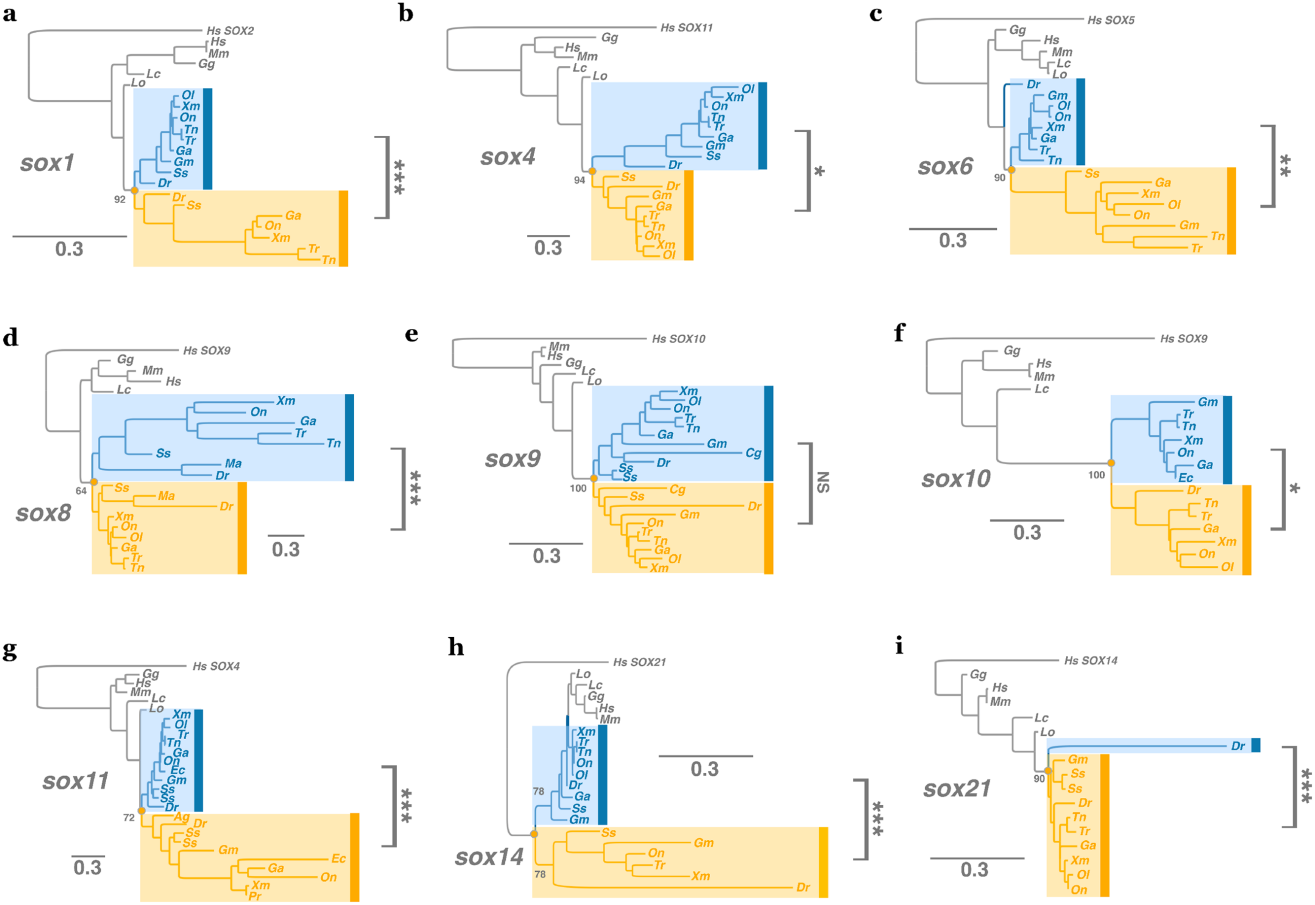
Phylogeny analyses of duplicated *sox* genes in teleosts. (a-i) Phylogenetic reconstructions were done for the nine genes *sox1*, *sox4*, *sox6*, *sox8*, *sox9*, *sox10*, *sox11*, *sox14* and *sox21*. Teleost-specific whole genome duplication paralogs are named *soxa* in blue and *soxb* in orange. Non-teleost orthologs are represented in grey. The most closely related human *SOX* gene was used to root the tree. Significant asymmetric evolution between *sox* paralogs in teleosts is highlighted on the phylogeny (see S4 Fig for details) using: NS for non significant, * for significant (P < 5%), ** for highly significant (P < 1%), and *** for extremely significant (P < 0.1%). Phylogenies were computed using PhyML and based on complete protein sequences alignment from mouse *M*. *musculus* (*Mm*), human *H*. *sapiens* (*Hs*), chicken *G*. *gallus* (*Gg*), coelacanth *L*. *chalumnae* (*Lc*), spotted gar *L*. *oculatus* (*Lo*), zebrafish *D*. *rerio* (*Dr*), catfish *C*. *gariepinus* (*Cg*), mud loach *M*. *anguillicaudatus* (*Ma*), salmon *S*. *salar* (*Ss*), cod *G*. *morhua* (*Gm*), tilapia *O*. *niloticus* (*On*), medaka *O*. *latipes* (*Ol*), platyfish *X*. *maculatus* (*Xm*), guppy *P*. *reticulata* (*Pr*), tetraodon *T*. *nigroviridis* (*Tn*), fugu *T*. *rubripes* (*Tr*) and stickleback *G*. *aculeatus* (*Ga*).

As described previously (Fig 1), more than half of *sox* genes are duplicated in teleostean fishes. However, duplicated genes can arise through small-scale duplication (SSD) or whole genome duplication (WGD). We determined, thanks to synteny and phylogeny analyses, that except for one specific case (*sox17/32*, see S2 Fig), all *sox* duplicates present in teleostean genomes originated from the teleost-specific WGD (Figs 2 and 3, and S1 Fig). Within each species, most duplicated *sox* genes are localized on ohnolog chromosomes, *i*.*e*. paralog chromosomes derived from WGD (Fig 2; [10, 61]). Furthermore, phylogenetic reconstructions showed that both paralogs are derived from a duplication event that occurred in the common ancestor of all studied teleost species (Fig 3). On the contrary, the so-called *sox32* gene in teleosts is a duplicate of *sox17* that probably arose through SSD: both genes are localized on the same chromosome (S2 Fig). The identification of *sox17/32* in all teleost genomes suggests that the event of SSD took place in a common ancestor of the analyzed species. To conclude, in teleost genomes, 11 *sox* genes are present as duplicates, 10 of which are derived from the teleost-specific WGD, showing the important role of this event in the expansion of the *sox* gene family in this lineage.

### Evolutionary fate of teleost-specific whole genome duplicated *sox* genes

Comparative analysis of the *sox* gene content in multiple teleost genomes allowed us to infer the evolutionary fate of WGD-derived *sox* duplicates in this lineage. On the 10 *sox* genes duplicated through the teleost-specific WGD (*sox1*, *4*, *6*, *8*, *9*, *10*, *11*, *14*, *19* and *21*, Fig 1), 20% (2/10, *sox4* and *sox9*) remained duplicated in all analyzed species, while the other 80% (8/10) showed recurrent loss in several but not all analyzed species. Of note, synteny analyses tend to show that it is always the same copy that has been lost. Likewise, considering the 6 *sox* genes (*sox2*, *3*, *5*, *7*, *13* and *18*, Fig 1) present as singleton in all teleostean species analyzed (the WGD specific to salmonids is not taking into account in this analysis), we observed that the same copy (*i*.*e*. the same ortholog) has been kept after the WGD (no reciprocal gene loss) (S3 Fig). The most parsimonious explanation for such an observation is that gene loss occurred after the teleost-specific WGD in a common ancestor of these species. Using dN/dS ratio-based analyses, we tested whether teleost-specific paralogs evolved differently than non-teleost orthologs, and whether they evolved differently from each other in post-WGD species (Fig 3, S4 Fig). Two groups of duplicated *sox* were observed: 1) *sox9* paralogs present similar coding sequence divergence but a significantly higher dN/dS ratio average than their non-teleost orthologs (model B: 0.032 versus 0.02), suggesting a relaxed purifying selection on both paralogs after duplication; 2) for all other duplicated *sox* genes (1, 4, 6, 8, 10, 11 14 and 21), the two paralogs evolved differently: one copy has evolved under strict evolutionary constraints leading to its conservation in all analyzed teleosts, whereas the second copy has evolved faster and is characterized by a higher dN/dS ratio (S4 Fig, model C, ω^a^ versus ω^b^), suggesting a relaxation of the purifying selection that led, in some species, to its loss. We hypothesize that the preservation of the “relaxed” copy in some species could be due to the acquisition of new properties (in term of gene expression and/or protein function) thanks to advantageous mutations, a mechanism also known as neo-functionalization [14, 62, 63].

### Evolution of conserved non-coding elements in the vicinity of WG-duplicated *sox* genes in teleosts

**Fig 4:**
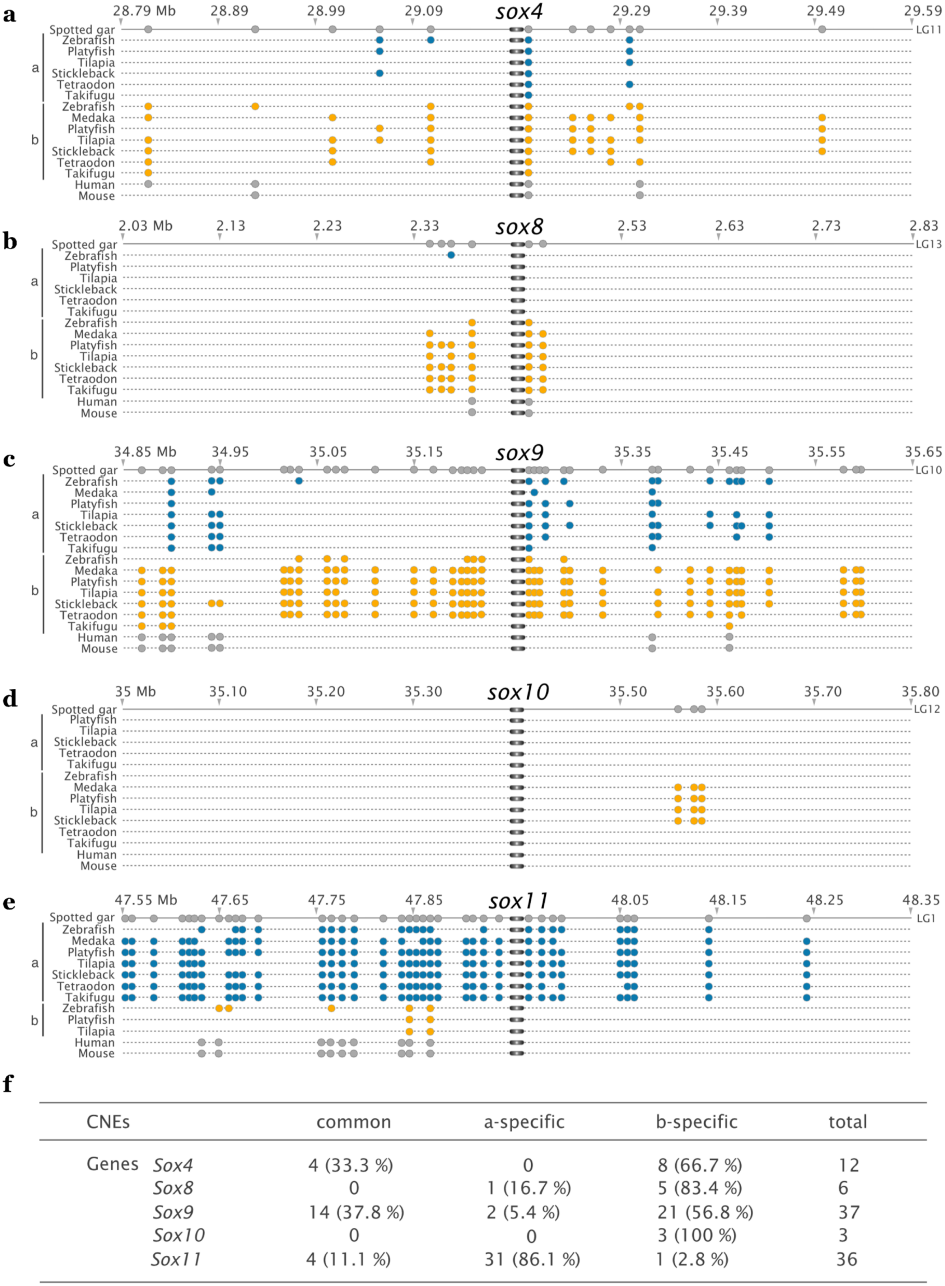
Conserved non-coding elements associated to *sox4*, *sox8*, *sox9*, *sox10* and *sox11*. Conserved non-coding elements (CNEs) are highlighted in the vicinity of fish *sox* genes. The spotted gar has been used as a reference. Blue and orange circles represent CNEs in the vicinity of WGD-derived paralogs a and b of *sox4* (a), *sox8* (b), *sox9* (c), *sox10* (d) and *sox11* (e). Grey circles are used for spotted gar, human and mouse CNEs. (f) Numbered representation of panels a to e above. *Sox11b* of the stickleback is not included in this study as there are no enough sequences available around the gene.

We compared the genomic environment of five *sox* duplicates arising from the teleost-specific WGD (*sox4*, *sox8*, *sox9*, *sox10 and sox11*) in different teleost species with their orthologous regions in the spotted gar and two tetrapods (human and mouse) in order to search for conserved non-coding elements (CNEs, Fig 4). We considered CNEs found only in actinopterygians, but also those that are present in tetrapods. CNEs localized in open reading frames or in UTRs have been excluded. The fact that CNEs are highly conserved through distant species implies that they are under functional constraints, suggesting an important role for the organism. For example, they might act as critical regions of regulatory control for their associated genes.

This analysis was done in order to compare the evolutionary forces acting on CNEs and neighbor genes after a WGD event. The number of CNEs detected varied widely according to the type of *sox* gene considered: only few CNEs were detected in the neighborhood of *sox8* and *sox10* (6 and 3 respectively, Fig 4f), whereas many more were identified in the vicinity of *sox4* (12, Fig 4f), and even more in the environment of *sox9* and *sox11* (37 and 36, respectively, Fig 4f). The degree of CNE conservation through vertebrates is also variable: around *sox8* and *sox11*, 33% and 25%, respectively, of the CNEs identified are shared by tetrapods and at least one of the two fish paralogs (Fig 4c and 4b), whereas none of the CNEs characterized in the vicinity of *sox10* were detected in the two tetrapod species used (Fig 4e). This analysis also showed that CNEs identified in the spotted gar tend to be asymmetrically distributed between the two teleostean paralogs, leading to the enrichment in putative ancestral regulatory element of one copy. For example, 66.7% (8/12, Fig 4f) of the CNEs detected in the vicinity of *sox4* in the spotted gar are found only around paralog b in teleosts. Similarly, 86.1%, 83.4%, 56.8% and 100% of the total CNEs detected in the environment of *sox11*, *sox8*, *sox9* and *sox10*, respectively, in the spotted gar are conserved in the environment of only one of the two paralog in teleosts (Fig 4f). This observed partition of the CNEs between paralogs is highly conserved through the species analyzed, suggesting an ancestral asymmetric distribution and indicating that, after the WGD event, one paralog evolved under strict evolutionary constraints in its non-coding environment whereas the second experienced a relaxed selection in the corresponding regions. An ancestral relaxation of selection on both paralogs, suggested by the distribution of highly conserved CNEs (found in tetrapods and spotted gar) between the two paralogs, could correspond to the first step facilitating the sub-functionalization process. Then, a subsequent asymmetric pressure of selection acting on the CNEs (suggested by the asymmetric distribution of CNEs between paralogs) could lead to lineage-specific combination of sub- and neo-functionalization of paralogs.

### Embryonic expression of duplicated *sox* genes in the zebrafish and the medaka

**Fig 5:**
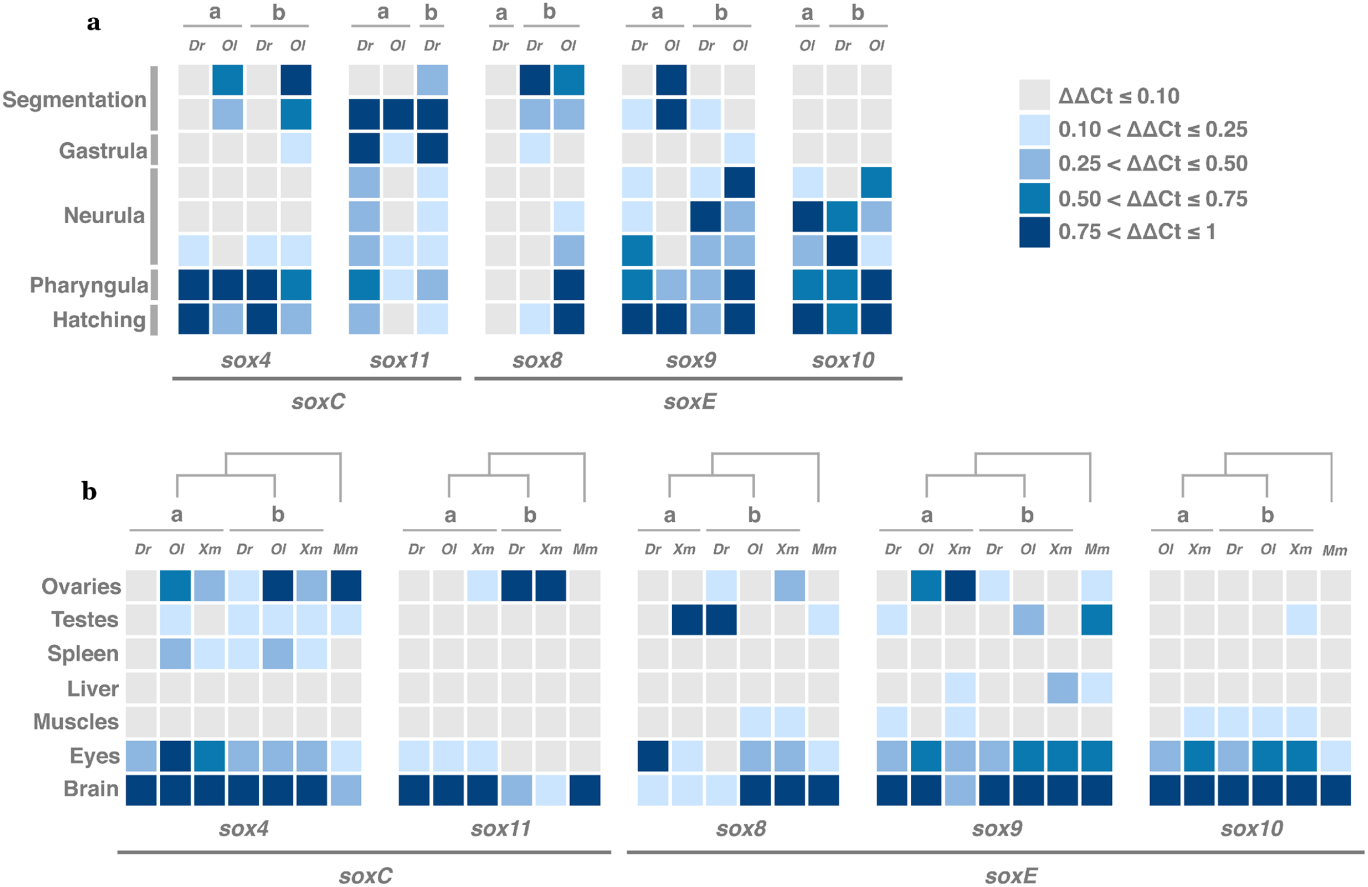
Expression patterns of *sox4*, *sox8*, *sox9*, *sox10* and *sox11* in three teleost species. (a) Expression patterns during *D*. *rerio* (*Dr*) and *O*. *latipes* (*Ol*) embryonic development. The five major developmental periods segmentation, gastrula, neurula, pharyngula and hatching are indicated on the left. (b) Expression patterns in seven adult tissues of *D*. *rerio* (*Dr*), *O*. *latipes* (*Ol*), *X*. *maculatus* (*Xm*) and *M*. *musculus* (*Mm*). qRT-PCR experiments were performed during embryonic development and adulthood. Data were normalized with the two housekeeping genes *bActin2* and *rpl7* and analyzed by the ΔΔCT method (Livak and Schmittgen 2001). Low expressed genes are indicated in grey (ΔΔCT≤ 0.10). More expressed genes are indicated in blue, the intensity of the blue increasing with the intensity of expression as indicated on the figure.

We analyzed the expression pattern of five duplicated *sox* genes (*sox4*, *sox8*, *sox9*, *sox10* and *sox11*) during embryogenesis in the zebrafish *Danio rerio* (*Dr*) and the medaka *Oryzias latipes* (*Ol*). We performed qRT-PCR experiments on eight developmental stages representing five crucial periods of the embryonic development: segmentation, gastrula, neurula, pharyngula and hatching [48, 49] (Fig 5a). During zebrafish development, we detected no strong differences of temporal expression pattern between *sox4*, *sox9* and *sox11* paralogs (Fig 5a). However, we noticed an early segmentation-specific expression of *sox11b*, and differences in expression level between *sox9* duplicates: *sox9a* is highly expressed during the pharyngula and the hatching stages, while *sox9b* is mainly expressed during the neurula stage. On the contrary, *sox8* paralogs exhibit a completely different expression pattern: *sox8b* is mainly expressed at the early stage of embryogenesis with a decreasing level of expression until the end of the gastrula stage, while no expression was detected for *sox8a* at any analyzed stages. This result suggests that the retention of *sox8a* in the zebrafish is probably not associated with a developmental function. During medaka embryogenesis, *sox4* and *sox10* duplicates present similar expression profiles (Fig 5a). In contrast, *sox9a* is mainly expressed during segmentation and hatching periods, whereas *sox9b* expression is spread from the gastrula stage to hatching, suggesting a differential evolution of the expression pattern of *sox9* paralogs in the medaka after the WGD event. If we compare *sox4* and *sox9* that are duplicated in both zebrafish and medaka, as shown Fig 5a, even if the expression profiles of both orthologs are overlapping, lineage-specific expression patterns are also detected: *sox4a*, *sox4b* and *sox9a* are highly expressed during segmentation in the medaka embryo only. For *sox10* and *sox11*, duplicated in medaka and zebrafish genomes, respectively, the combined expression pattern of both paralogs in one species is similar to the corresponding singleton in the other species (Fig 5a). The case of *sox8*, duplicated in the zebrafish genome, is particular: *sox8a* is not expressed during zebrafish development and the expression profiles of *sox8b* in both species are completely different: *sox8b* is expressed from segmentation to gastrula stages in the zebrafish embryo, while the medaka *sox8b* is expressed during segmentation and then highly during pharyngula and hatching periods. These results indicate that duplicated *sox* genes show overlapping/redundant expression during embryogenesis in zebrafish and medaka. However, we noticed examples of strong divergence in favor of a lineage-specific evolution of orthologs expression during embryogenesis.

### Expression of duplicated *sox* genes in adult fish

Three fish species (the zebrafish, the medaka and the platyfish *Xiphophorus maculatus Xm*), and a tetrapod (the mouse *Mus musculus Mm*) have been used. Six different male and female adult tissues were collected and tested: gonads (ovaries/testes), spleen, liver, muscles, eyes and brain (Fig 5b). We analyzed the expression pattern of the five duplicated genes *sox4*, *sox8*, *sox9*, *sox10* and *sox11*. Except for *sox4* duplicates in medaka, for which patterns of expression are equivalent, all other studied *sox* duplicates show, in the three fish species, differences in their expression during adulthood. 50% of the duplicates exhibit overlapping expression in some tissues, with one paralog expressed in at least one additional tissue. For instance, in platyfish, *sox10a* and *sox10b* are both expressed in muscles, eyes and brain, and *sox10b* is, in addition, detected in testes. This pattern is also observed for *sox10* and *sox4* duplicates in medaka and zebrafish respectively, and for *sox4*, *sox11* and *sox9* duplicates in platyfish. The other 50% of *sox* duplicates present overlapping profiles of expression in some tissues, and each paralog is also expressed specifically in one or more tissues. For example, in the platyfish, *sox8a* and *sox8b* are both expressed in eyes and brain, but also in testes and ovaries, respectively. Similarly, in the medaka, *sox9a* and *sox9b* are detected in ovaries and testes respectively, whereas both are expressed in brain and eyes. The same situation is observed for *sox8*, *sox9* and *sox11* duplicates in zebrafish. These results demonstrate that, after an event of genome duplication, the evolutionary retention of each paralogs led to specific pattern of tissue-specific transcriptional divergence.

Besides, the comparison of the expression patterns in fish with those of the corresponding orthologs in mouse allowed us to infer the putative expression pattern of the ancestral gene in the common ancestor of teleosts and tetrapods. This approach could help to identify potential cases of specialization, sub-functionalization and/or neo-functionalization that followed the teleost-specific WGD. For example, *sox11a* and *sox11b* in teleosts are expressed in brain and ovaries, while *sox11* in mouse is only detected in brain, suggesting both that the ancestral *sox11* gene was only expressed in brain and that the ovary-specific expression in teleost could be due to the acquisition of a new pattern of expression. Nonetheless, we can’t exclude the fact that *sox11* expression in ovaries has been lost in the mouse lineage, or under the threshold of detection in our experimental conditions. Same analyses/hypotheses could be done if we consider the teleost-specific expression of *sox4* in the spleen, *sox11* in the eyes, *sox8* in muscles and ovaries, *sox9* in muscles and *sox10* in muscles and testes, but more experiments using more species of tetrapods are necessary to correctly assess this question. Overall, this study highlights lineage-specific cases of transcriptional divergence, either between distant species (medaka and zebrafish for example), or between more closely related species (medaka and platyfish in this study).

## Discussion

### Expansion of the *sox* family after the teleost-specific whole genome duplication

This study highlights an important expansion of the *sox* transcription factor gene family in teleostean fishes. Thanks to synteny and phylogeny analyses, we demonstrated that this enlargement is the consequence of the well-established teleost-specific WGD estimated to have occurred around 226–316 mya [6, 9, 10]. It has been previously suggested that four pairs of *sox* genes (*sox9*, *sox10*, *sox11* and *sox19*) were derived from the teleost-specific WGD [29, 37, 38, 41]. In this study, we demonstrated that six pairs more arose from this WGD event. Strikingly, the observed retention rate of *sox* duplicates (52.6%, 10/19) following the WGD is higher than the already described average of global gene retention of 12 to 24% deduced from other genome-wide analyses in teleosts [9, 16, 61, 64, 65]. This observation confirms the hypothesis suggesting the preferential retention of specific gene categories after WGDs such as genes encoding for transcription factors [16, 64, 66, 67]. Accordingly, the retention rate of *sox* duplicates in the salmon *Salmo salar* and the rainbow trout *Oncorhynchus mykiss* after the additional and more recent WGD event that occurred in salmonidae 100 mya [60] is higher than 80% (Fig 1). This result is in agreement with the hypothesis suggesting that salmonidae genomes are still in the stage of re-diploidization process and consequently still contain a high number of paralogs. Finally, this study indicates that *sox* paralogs derived from small-scale duplication (SSD) are rare in the teleost lineage. Indeed, only one case has been characterized: *sox17* and *sox32* detected in all the analyzed teleosts genomes are SSD-derived paralogs. The fact that *sox* transcription factors expand mainly by WGDs rather than SSDs might be linked to dosage-balanced sensitivity: changes in their relative quantity of protein are deleterious [68]. Consequently, the expansion of *sox* genes in stem vertebrates, as many other transcription factor families, seems mainly due to the two WGDs that occurred at the base of the vertebrate lineage [37, 69, 70]. Interestingly, some authors argued that WGD events might allow and favor genetic innovations and diversification [5, 18]. Transposed to *sox* genes, this idea implies that the expansion of the *sox* family after the teleost-specific WGD, and the subsequent high retention rate of paralogs (providing raw material for evolutionary novelties), might have been key events in teleost diversification [32].

### Evolution of duplicated *sox* genes and related CNEs in teleosts

This study, based on comparative genomics analyses, shows that, after an event of WGD, the copy of a gene that is conserved in all species has evolved under strong selective constraints, highlighted by few changes in its sequence. On the contrary, the second copy of the same gene, which is not obligatory still present in all genomes, has probably experienced a relaxation of selective constraints, allowing changes in the sequence, function evolution and subsequent evolutionary diversification. Such an asymmetric evolution in terms of selective constraints after an event of WGD has been already reported in several studies [16, 71–75]. Of note, the relative contribution of both relaxed negative selection and positive selection in this asymmetric evolution is rather difficult to estimate due to the ancientness of the teleost-specific WGD and to the motley pathways of evolution of paralogs over several hundreds of millions of years [18, 25, 74, 76, 77]. Furthermore, it seems that there is a correlation between the retention rate and the divergence of two paralogs. On the 10 WG-duplicated *sox* genes in teleostean genomes, 2 are duplicated in all species analyzed: *sox4* and *sox9*, of which paralogs a and b possess closely related and low dN/dS ratios. This observation can be explained if we consider a rapid sub-functionalization event just after the WGD. If so, the maintenance of the 2 paralogs is then absolutely necessary to insure the ancestral function of the gene and the survival of the organism. On the contrary, genes presenting paralogs with a more divergent evolution, at the molecular level, are more often detected as singleton.

We also looked at the genomic environment of *sox* genes, leading to the identification of CNEs that correspond to highly conserved non-coding sequences. Even if sequence similarities between divergent organisms imply functional constraint, the precise mode of action and function of the vast majority of CNEs identified remain a mystery, but, whatever their functioning, it is accepted that CNEs represent critical regulatory elements being part of gene regulatory networks that define the tight and accurate regulation of genes. In tetrapods, CNEs seem to be highly constrained whereas, in teleost, they appear to be under relaxed evolution leading to their rapid divergence, both within teleost species and in comparison to their tetrapods’ orthologs [9, 78, 79]. It seems that the teleost-specific WGD could be a reason explaining the elevated rate of evolution of CNEs in this lineage [80, 81]. However, previous publications concerning CNEs evolution considered CNEs alone, and did not take into account neighbor genes that are potentially target of regulation of proximal CNEs. In this study, we decided to analyze CNEs as being part of an entity comprising coding and non-coding sequences (*i*.*e*. genes and surrounding CNEs), and we observed a correspondence between the distribution of CNEs around paralogs and the molecular evolution of their coding sequences. Indeed, when the asymmetric divergence between paralogs is marked, we notice a tendency of CNEs to be conserved with the most constrained ones. Hence, paralogs *sox4b*, *sox8b* and *sox11a* (Fig 3b, 3d and 3g) possess 66.7%, 83.4% and 86.1%, respectively, of the identified CNEs (Fig 4f). Even more striking, if we look solely at CNEs conserved through teleost and tetrapods (defined below as vCNEs), considered as highly important ones due to their ancientness, we also remark that they are systematically found in the vicinity of less divergent paralogs as 2, 2 and 5 vCNEs are identified around *sox4b*, *sox8b* and *sox11a*, respectively.

More generally, it seems that coding and adjacent non-coding regions follow the same evolutionary trajectories. Consequently, we can consider it as a co-evolutionary unit in which regulatory elements and genes have a tendency to diverge, or not, together. Same results were obtained in a study analyzing HoxA clusters in teleost [75], but Hox clusters arose through tandem duplications, while in our study we look at genes and CNEs obtained after an event of WGD.

### Functional diversification of *sox* duplicates

Our results indicate lineage-specific divergent evolution of paralogs expression profiles after the teleost specific WGD as two paralogs of the same gene do not automatically show the same pattern of expression in at least two different species. For instance, species-specific differences are detected for *sox8a* between adult zebrafish and platyfish, and for *sox9a* and *sox9b* between adult zebrafish and medaka. These observations are in agreement with previous studies that infer species-specific sub-functionalization of *sox9* duplicates in teleosts [39, 42]. Interestingly, the medaka and the platyfish, that are more closely related species, also show specific-species differences in *sox* paralogs expression patterns as it is the case for *sox9b* which is expressed in liver, eyes and brain in the platyfish while its ortholog in the medaka is expressed in testes, eyes and brain. This work also underline that most of the *sox* paralogs remain partially redundant as some *sox* duplicates exhibit overlapping expression sites during embryogenesis and adulthood suggesting, besides specific expression patterns and function, similar biochemical and regulatory properties.

If we try to search for a correspondence between coding sequence divergence and transcriptional divergence of paralogs, the conclusion is that apparently there are no evident rules, since pairs of paralogs that have considerable coding sequence divergence did not automatically display more transcriptional divergence than pairs of paralogs that are similarly conserved in their coding sequence. Likewise, less divergent paralogs did not always show conserved transcriptional patterns. Similar observations have been done in a recent study looking at duplicated HIF factors in zebrafish [82]. So, it seems that, after the teleost-specific WGD, the evolutionary retention of some paralogs is due to the establishment of peculiar and lineage-specific couples of coding sequence divergence and adult tissue-specific or developmental-specific transcriptional divergence.

## Conclusion

This work demonstrates that all duplicated *sox* genes, except *sox17* paralogs, are derived from the teleost-specific WGD, and that genes and surrounding CNEs are co-evolutionary units, the evolution of which is driven by same pressures of selection. Regarding expression analysis experiments, no obvious rules could be deduced concerning a potential link between sequence and transcriptional divergences, as divergent molecular evolution between paralogs is not systematically associated to a divergence of the expression pattern. The observed lineage-specific evolution of paralogs at the molecular and expression levels is probably the consequences of lineage-specific sub- and/or neo-functionalization after the teleost-specific WGD. This phenomenon could have contributed to functional divergence among teleost and consequently could have promoted the genetic and phenotypic diversification observed currently in this lineage. As already mentioned, the co-evolution of coding and non-coding sequences suggests that relaxation in regulatory regions could be the first step facilitating relaxation in corresponding coding sequences.

## Supporting information

S1 Fig Macrosynteny analysis of teleost-specific whole genome duplicated *sox* genes. (a-i) Orthology relationships across four teleost species (*D*. *rerio* (*Dr*), *O*. *latipes* (*Ol*), *G*. *aculeatus* (Ga) and *T*. *nigroviridis* (*Tn*)) are represented. *Soxa* and *soxb* paralogs are respectively in blue and orange.
(PDF)

S2 Fig Case of *sox17/32* in teleostean fishes. a) Phylogeny analysis of *sox17/32*. The phylogeny was computed using PhyML and based on protein sequences alignment (*M*. *musculus* (*Mm*), *H*. *sapiens* (*Hs*), *G*. *gallus* (*Gg*), *L*. *chalumnae* (*Lc*), *L*. *oculatus* (*Lo*), *D*. *rerio* (*Dr*), *S*. *salar* (*Ss*), *O*. *niloticus* (*On*), *O*. *latipes* (*Ol*), *X*. *maculatus* (*Xm*), *T*. *nigroviridis* (*Tn*), *T*. *rubripes* (*Tr*) and *G*. *aculeatus* (*Ga*)). Non-teleost *sox17* orthologs are represented in grey. The tree is rooted with the human *SOX7*. b) Macrosynteny analysis of *sox17/32*. Orthology relationships across the four zebrafish *D*. *rerio* (*Dr*), medaka *O*. *latipes* (*Ol*), stickleback *G*. *aculeatus (Ga)* and Tetraodon *T*. *nigroviridis* (*Tn*) are represented. c) Microsynteny analysis of *sox17*/*32*. *sox17*/*32*-containing region in zebrafish, medaka, stickleback, and tetraodon genomes have been analyzed. *Sox17* and *sox32* are highlighted in light blue and dark blue respectively.
(PDF)

S3 Fig Orthology relationships of *sox2*, *sox3*, *sox5*, *sox7*, *sox13* and *sox18*. The four genomes of *D*. *rerio (Dr)*, *O*. *latipes (Ol)*, *G*. *aculeatus (Ga) and T*. *nigroviridis (Tn)* have been used. Grey lines connect orthologous genes on the different chromosomes (Chr) or linkage-group (Gr). The *sox* gene considered is highlighted in dark grey. a) *sox2*, b) *sox3*, c) *sox5*, d) *sox7*, e) *sox13* and f) *sox18*.
(PDF)

S4 Fig dN/dS ratio analyses of WGD-derived *sox* paralogs in teleost. Log likelihood values (LnL) are obtained for each model (A, B or C). Parameters ω^r^, ω^p^, ω^a^ and ω^b^ are the dN/dS ratios for all branches, both paralogs branches, paralogs a branches and paralogs b branches, respectively. Tests were done by comparing twice the difference of likelihood values to a X^2^ distribution with degrees of freedom (df) equal to the difference in number of free parameters of each model.
(PDF)

S5 Fig Student’s t-test to support the hypothesis of asymmetry of CNEs number. We performed a student’s t-test based on the difference between means of the number of CNEs in the respective environment of the two paralogs.
(PDF)

S1 Table Accession numbers or scaffold references of the sequences used in this study. Accession numbers or scaffold references of the *sox* sequences are indicated in yellow and pink boxes for non-teleost and teleost species respectively. They are organized in *sox* group. Boxes are grey when no corresponding *sox* sequences were detected.
(XLS)

## References

[1] Santos ME, Salzburger W. Evolution. How cichlids diversify. Science. 2012;338(6107):619–21. Epub 2012/11/03. doi: 10.1126/science.1224818 . 23118176 10.1126/science.1224818

[2] Haldane JB. A Mathematical Theory of Natural and Artificial Selection Part X. Some Theorems on Artificial Selection. Genetics. 1934;19(5):412–29. Epub 1934/09/01. ; 17246731 10.1093/genetics/19.5.412 PMC1208491

[3] Kaessmann H. Origins, evolution, and phenotypic impact of new genes. Genome research. 2010;20(10):1313–26. Epub 2010/07/24. doi: 10.1101/gr.101386.109 ; 20651121 10.1101/gr.101386.109 PMC2945180

[4] Ohno S, Wolf U, Atkin NB. Evolution from fish to mammals by gene duplication. Hereditas. 1968;59(1):169–87. Epub 1968/01/01. . 5662632 10.1111/j.1601-5223.1968.tb02169.x

[5] Freeling M, Thomas BC. Gene-balanced duplications, like tetraploidy, provide predictable drive to increase morphological complexity. Genome research. 2006;16(7):805–14. Epub 2006/07/05. doi: 10.1101/gr.3681406 . 16818725 10.1101/gr.3681406

[6] Van de Peer Y, Maere S, Meyer A. The evolutionary significance of ancient genome duplications. Nature reviews Genetics. 2009;10(10):725–32. Epub 2009/08/05. doi: 10.1038/nrg2600 . 19652647 10.1038/nrg2600

[7] Dehal P, Boore JL. Two rounds of whole genome duplication in the ancestral vertebrate. PLoS biology. 2005;3(10):e314 Epub 2005/09/01. doi: 10.1371/journal.pbio.0030314 ; 16128622 10.1371/journal.pbio.0030314 PMC1197285

[8] Amores A, Force A, Yan YL, Joly L, Amemiya C, Fritz A, et al Zebrafish hox clusters and vertebrate genome evolution. Science. 1998;282(5394):1711–4. Epub 1998/11/30. . 9831563 10.1126/science.282.5394.1711

[9] Jaillon O, Aury JM, Brunet F, Petit JL, Stange-Thomann N, Mauceli E, et al Genome duplication in the teleost fish Tetraodon nigroviridis reveals the early vertebrate proto-karyotype. Nature. 2004;431(7011):946–57. Epub 2004/10/22. doi: 10.1038/nature03025 . 15496914 10.1038/nature03025

[10] Kasahara M, Naruse K, Sasaki S, Nakatani Y, Qu W, Ahsan B, et al The medaka draft genome and insights into vertebrate genome evolution. Nature. 2007;447(7145):714–9. Epub 2007/06/08. doi: 10.1038/nature05846 . 17554307 10.1038/nature05846

[11] Otto SP. The evolutionary consequences of polyploidy. Cell. 2007;131(3):452–62. Epub 2007/11/06. doi: 10.1016/j.cell.2007.10.022 . 17981114 10.1016/j.cell.2007.10.022

[12] Wolfe KH. Yesterday's polyploids and the mystery of diploidization. Nature reviews Genetics. 2001;2(5):333–41. Epub 2001/05/02. doi: 10.1038/35072009 . 11331899 10.1038/35072009

[13] Semon M, Wolfe KH. Rearrangement rate following the whole-genome duplication in teleosts. Molecular biology and evolution. 2007;24(3):860–7. Epub 2007/01/16. doi: 10.1093/molbev/msm003 . 17218642 10.1093/molbev/msm003

[14] Lynch M, Conery JS. The evolutionary fate and consequences of duplicate genes. Science. 2000;290(5494):1151–5. Epub 2000/11/10. . 11073452 10.1126/science.290.5494.1151

[15] Bowers JE, Chapman BA, Rong J, Paterson AH. Unravelling angiosperm genome evolution by phylogenetic analysis of chromosomal duplication events. Nature. 2003;422(6930):433–8. Epub 2003/03/28. doi: 10.1038/nature01521 . 12660784 10.1038/nature01521

[16] Brunet FG, Roest Crollius H, Paris M, Aury JM, Gibert P, Jaillon O, et al Gene loss and evolutionary rates following whole-genome duplication in teleost fishes. Molecular biology and evolution. 2006;23(9):1808–16. Epub 2006/07/01. doi: 10.1093/molbev/msl049 . 16809621 10.1093/molbev/msl049

[17] Kellis M, Birren BW, Lander ES. Proof and evolutionary analysis of ancient genome duplication in the yeast Saccharomyces cerevisiae. Nature. 2004;428(6983):617–24. Epub 2004/03/09. doi: 10.1038/nature02424 . 15004568 10.1038/nature02424

[18] Conant GC, Wolfe KH. Turning a hobby into a job: how duplicated genes find new functions. Nature reviews Genetics. 2008;9(12):938–50. Epub 2008/11/19. doi: 10.1038/nrg2482 . 19015656 10.1038/nrg2482

[19] Semon M, Wolfe KH. Consequences of genome duplication. Current opinion in genetics & development. 2007;17(6):505–12. Epub 2007/11/17. doi: 10.1016/j.gde.2007.09.007 . 18006297 10.1016/j.gde.2007.09.007

[20] Volff JN. Genome evolution and biodiversity in teleost fish. Heredity. 2005;94(3):280–94. Epub 2005/01/28. doi: 10.1038/sj.hdy.6800635 . 15674378 10.1038/sj.hdy.6800635

[21] De Bodt S, Maere S, Van de Peer Y. Genome duplication and the origin of angiosperms. Trends in ecology & evolution. 2005;20(11):591–7. Epub 2006/05/17. doi: 10.1016/j.tree.2005.07.008 . 16701441 10.1016/j.tree.2005.07.008

[22] Magallon S, Castillo A. Angiosperm diversification through time. American journal of botany. 2009;96(1):349–65. Epub 2009/01/01. doi: 10.3732/ajb.0800060 . 21628193 10.3732/ajb.0800060

[23] Soltis DE, Albert VA, Leebens-Mack J, Bell CD, Paterson AH, Zheng C, et al Polyploidy and angiosperm diversification. American journal of botany. 2009;96(1):336–48. Epub 2009/01/01. doi: 10.3732/ajb.0800079 . 21628192 10.3732/ajb.0800079

[24] Soltis PS, Soltis DE. Flower diversity and angiosperm diversification. Methods Mol Biol. 2014;1110:85–102. Epub 2014/01/08. doi: 10.1007/978-1-4614-9408-9_4 . 24395253 10.1007/978-1-4614-9408-9_4

[25] Chain FJ, Evans BJ. Multiple mechanisms promote the retained expression of gene duplicates in the tetraploid frog Xenopus laevis. PLoS genetics. 2006;2(4):e56 Epub 2006/05/10. doi: 10.1371/journal.pgen.0020056 ; 16683033 10.1371/journal.pgen.0020056 PMC1449897

[26] Chain FJ, Ilieva D, Evans BJ. Duplicate gene evolution and expression in the wake of vertebrate allopolyploidization. BMC evolutionary biology. 2008;8:43 Epub 2008/02/12. doi: 10.1186/1471-2148-8-43 ; 18261230 10.1186/1471-2148-8-43 PMC2275784

[27] Semon M, Wolfe KH. Preferential subfunctionalization of slow-evolving genes after allopolyploidization in Xenopus laevis. Proceedings of the National Academy of Sciences of the United States of America. 2008;105(24):8333–8. Epub 2008/06/11. doi: 10.1073/pnas.0708705105 ; 18541921 10.1073/pnas.0708705105 PMC2448837

[28] Ahn D, You KH, Kim CH. Evolution of the tbx6/16 subfamily genes in vertebrates: insights from zebrafish. Molecular biology and evolution. 2012;29(12):3959–83. Epub 2012/08/24. doi: 10.1093/molbev/mss199 . 22915831 10.1093/molbev/mss199

[29] Braasch I, Brunet F, Volff JN, Schartl M. Pigmentation pathway evolution after whole-genome duplication in fish. Genome biology and evolution. 2009;1:479–93. Epub 2009/01/01. doi: 10.1093/gbe/evp050 ; 20333216 10.1093/gbe/evp050 PMC2839281

[30] Opazo JC, Butts GT, Nery MF, Storz JF, Hoffmann FG. Whole-genome duplication and the functional diversification of teleost fish hemoglobins. Molecular biology and evolution. 2013;30(1):140–53. Epub 2012/09/06. doi: 10.1093/molbev/mss212 ; 22949522 10.1093/molbev/mss212 PMC3525417

[31] Bowles J, Schepers G, Koopman P. Phylogeny of the SOX family of developmental transcription factors based on sequence and structural indicators. Developmental biology. 2000;227(2):239–55. Epub 2000/11/10. doi: 10.1006/dbio.2000.9883 . 11071752 10.1006/dbio.2000.9883

[32] Sarkar A, Hochedlinger K. The sox family of transcription factors: versatile regulators of stem and progenitor cell fate. Cell stem cell. 2013;12(1):15–30. Epub 2013/01/08. doi: 10.1016/j.stem.2012.12.007 ; 23290134 10.1016/j.stem.2012.12.007 PMC3608206

[33] Gubbay J, Collignon J, Koopman P, Capel B, Economou A, Munsterberg A, et al A gene mapping to the sex-determining region of the mouse Y chromosome is a member of a novel family of embryonically expressed genes. Nature. 1990;346(6281):245–50. Epub 1990/07/19. doi: 10.1038/346245a0 . 2374589 10.1038/346245a0

[34] Sinclair AH, Berta P, Palmer MS, Hawkins JR, Griffiths BL, Smith MJ, et al A gene from the human sex-determining region encodes a protein with homology to a conserved DNA-binding motif. Nature. 1990;346(6281):240–4. Epub 1990/07/19. doi: 10.1038/346240a0 . 1695712 10.1038/346240a0

[35] Hawkins JR. Mutational analysis of SRY in XY females. Human mutation. 1993;2(5):347–50. Epub 1993/01/01. doi: 10.1002/humu.1380020504 . 8257986 10.1002/humu.1380020504

[36] Phochanukul N, Russell S. No backbone but lots of Sox: Invertebrate Sox genes. The international journal of biochemistry & cell biology. 2010;42(3):453–64. Epub 2009/07/11. doi: 10.1016/j.biocel.2009.06.013 . 19589395 10.1016/j.biocel.2009.06.013

[37] Okuda Y, Yoda H, Uchikawa M, Furutani-Seiki M, Takeda H, Kondoh H, et al Comparative genomic and expression analysis of group B1 sox genes in zebrafish indicates their diversification during vertebrate evolution. Developmental dynamics: an official publication of the American Association of Anatomists. 2006;235(3):811–25. Epub 2006/01/13. doi: 10.1002/dvdy.20678 . 16408288 10.1002/dvdy.20678

[38] Chiang EF, Yan YL, Guiguen Y, Postlethwait J, Chung B. Two Cyp19 (P450 aromatase) genes on duplicated zebrafish chromosomes are expressed in ovary or brain. Molecular biology and evolution. 2001;18(4):542–50. Epub 2001/03/27. . 11264405 10.1093/oxfordjournals.molbev.a003833

[39] Cresko WA, Yan YL, Baltrus DA, Amores A, Singer A, Rodriguez-Mari A, et al Genome duplication, subfunction partitioning, and lineage divergence: Sox9 in stickleback and zebrafish. Developmental dynamics: an official publication of the American Association of Anatomists. 2003;228(3):480–9. Epub 2003/10/28. doi: 10.1002/dvdy.10424 . 14579386 10.1002/dvdy.10424

[40] de Martino S, Yan YL, Jowett T, Postlethwait JH, Varga ZM, Ashworth A, et al Expression of sox11 gene duplicates in zebrafish suggests the reciprocal loss of ancestral gene expression patterns in development. Developmental dynamics: an official publication of the American Association of Anatomists. 2000;217(3):279–92. Epub 2000/03/31. doi: 10.1002/(SICI)1097-0177(200003)217:3<279::AID-DVDY6>3.0.CO;2-S . 10741422 10.1002/(SICI)1097-0177(200003)217:3<279::AID-DVDY6>3.0.CO;2-S

[41] Hoegg S, Brinkmann H, Taylor JS, Meyer A. Phylogenetic timing of the fish-specific genome duplication correlates with the diversification of teleost fish. Journal of molecular evolution. 2004;59(2):190–203. Epub 2004/10/16. doi: 10.1007/s00239-004-2613-z . 15486693 10.1007/s00239-004-2613-z

[42] Kluver N, Kondo M, Herpin A, Mitani H, Schartl M. Divergent expression patterns of Sox9 duplicates in teleosts indicate a lineage specific subfunctionalization. Development genes and evolution. 2005;215(6):297–305. Epub 2005/04/09. doi: 10.1007/s00427-005-0477-x . 15818483 10.1007/s00427-005-0477-x

[43] Koopman P, Schepers G, Brenner S, Venkatesh B. Origin and diversity of the SOX transcription factor gene family: genome-wide analysis in Fugu rubripes. Gene. 2004;328:177–86. Epub 2004/03/17. doi: 10.1016/j.gene.2003.12.008 . 15019997 10.1016/j.gene.2003.12.008

[44] Mavropoulos A, Devos N, Biemar F, Zecchin E, Argenton F, Edlund H, et al sox4b is a key player of pancreatic alpha cell differentiation in zebrafish. Developmental biology. 2005;285(1):211–23. Epub 2005/08/02. doi: 10.1016/j.ydbio.2005.06.024 . 16055112 10.1016/j.ydbio.2005.06.024

[45] Zhang L, Lin D, Zhang Y, Ma G, Zhang W. A homologue of Sox11 predominantly expressed in the ovary of the orange-spotted grouper Epinephelus coioides. Comparative biochemistry and physiology Part B, Biochemistry & molecular biology. 2008;149(2):345–53. Epub 2007/11/23. doi: 10.1016/j.cbpb.2007.10.006 . 18032080 10.1016/j.cbpb.2007.10.006

[46] Zhang L, Zhu T, Lin D, Zhang Y, Zhang W. A second form of Sox11 homologue identified in the orange-spotted grouper Epinephelus coioides: analysis of sequence and mRNA expression patterns. Comparative biochemistry and physiology Part B, Biochemistry & molecular biology. 2010;157(4):415–22. Epub 2010/09/21. doi: 10.1016/j.cbpb.2010.09.004 . 20851206 10.1016/j.cbpb.2010.09.004

[47] Nüsslein-Vollhard C D R. Zebrafish: a practical approach. Oxford University Press, USA 2002.

[48] Iwamatsu T. Stages of normal development in the medaka Oryzias latipes. Mechanisms of development. 2004;121(7–8):605–18. Epub 2004/06/24. doi: 10.1016/j.mod.2004.03.012 . 15210170 10.1016/j.mod.2004.03.012

[49] Kimmel CB, Ballard WW, Kimmel SR, Ullmann B, Schilling TF. Stages of embryonic development of the zebrafish. Developmental dynamics: an official publication of the American Association of Anatomists. 1995;203(3):253–310. Epub 1995/07/01. doi: 10.1002/aja.1002030302 . 8589427 10.1002/aja.1002030302

[50] Altschul SF, Gish W, Miller W, Myers EW, Lipman DJ. Basic local alignment search tool. Journal of molecular biology. 1990;215(3):403–10. Epub 1990/10/05. doi: 10.1016/S0022-2836(05)80360-2 . 2231712 10.1016/S0022-2836(05)80360-2

[51] Tamura K, Dudley J, Nei M, Kumar S. MEGA4: Molecular Evolutionary Genetics Analysis (MEGA) software version 4.0. Molecular biology and evolution. 2007;24(8):1596–9. Epub 2007/05/10. doi: 10.1093/molbev/msm092 . 17488738 10.1093/molbev/msm092

[52] Larkin MA, Blackshields G, Brown NP, Chenna R, McGettigan PA, McWilliam H, et al Clustal W and Clustal X version 2.0. Bioinformatics. 2007;23(21):2947–8. Epub 2007/09/12. doi: 10.1093/bioinformatics/btm404 . 17846036 10.1093/bioinformatics/btm404

[53] Thompson JD, Higgins DG, Gibson TJ. CLUSTAL W: improving the sensitivity of progressive multiple sequence alignment through sequence weighting, position-specific gap penalties and weight matrix choice. Nucleic acids research. 1994;22(22):4673–80. Epub 1994/11/11. ; 7984417 10.1093/nar/22.22.4673 PMC308517

[54] Galtier N, Gouy M, Gautier C. SEAVIEW and PHYLO_WIN: two graphic tools for sequence alignment and molecular phylogeny. Computer applications in the biosciences: CABIOS. 1996;12(6):543–8. Epub 1996/12/01. . 9021275 10.1093/bioinformatics/12.6.543

[55] Anisimova M, Gascuel O. Approximate likelihood-ratio test for branches: A fast, accurate, and powerful alternative. Systematic biology. 2006;55(4):539–52. Epub 2006/06/21. doi: 10.1080/10635150600755453 . 16785212 10.1080/10635150600755453

[56] Yang Z. PAML 4: phylogenetic analysis by maximum likelihood. Molecular biology and evolution. 2007;24(8):1586–91. Epub 2007/05/08. doi: 10.1093/molbev/msm088 . 17483113 10.1093/molbev/msm088

[57] Livak KJ, Schmittgen TD. Analysis of relative gene expression data using real-time quantitative PCR and the 2(-Delta Delta C(T)) Method. Methods. 2001;25(4):402–8. Epub 2002/02/16. doi: 10.1006/meth.2001.1262 . 11846609 10.1006/meth.2001.1262

[58] Graves JA. Sex chromosome specialization and degeneration in mammals. Cell. 2006;124(5):901–14. Epub 2006/03/15. doi: 10.1016/j.cell.2006.02.024 . 16530039 10.1016/j.cell.2006.02.024

[59] Amores A, Catchen J, Ferrara A, Fontenot Q, Postlethwait JH. Genome evolution and meiotic maps by massively parallel DNA sequencing: spotted gar, an outgroup for the teleost genome duplication. Genetics. 2011;188(4):799–808. Epub 2011/08/11. doi: 10.1534/genetics.111.127324 ; 21828280 10.1534/genetics.111.127324 PMC3176089

[60] Berthelot C, Brunet F, Chalopin D, Juanchich A, Bernard M, Noel B, et al The rainbow trout genome provides novel insights into evolution after whole-genome duplication in vertebrates. Nature communications. 2014;5:3657 Epub 2014/04/24. doi: 10.1038/ncomms4657 ; 24755649 10.1038/ncomms4657 PMC4071752

[61] Woods IG, Wilson C, Friedlander B, Chang P, Reyes DK, Nix R, et al The zebrafish gene map defines ancestral vertebrate chromosomes. Genome research. 2005;15(9):1307–14. Epub 2005/08/20. doi: 10.1101/gr.4134305 ; 16109975 10.1101/gr.4134305 PMC1199546

[62] He X, Zhang J. Rapid subfunctionalization accompanied by prolonged and substantial neofunctionalization in duplicate gene evolution. Genetics. 2005;169(2):1157–64. Epub 2005/01/18. doi: 10.1534/genetics.104.037051 ; 15654095 10.1534/genetics.104.037051 PMC1449125

[63] Rastogi S, Liberles DA. Subfunctionalization of duplicated genes as a transition state to neofunctionalization. BMC evolutionary biology. 2005;5:28 Epub 2005/04/16. doi: 10.1186/1471-2148-5-28 ; 15831095 10.1186/1471-2148-5-28 PMC1112588

[64] Kassahn KS, Dang VT, Wilkins SJ, Perkins AC, Ragan MA. Evolution of gene function and regulatory control after whole-genome duplication: comparative analyses in vertebrates. Genome research. 2009;19(8):1404–18. Epub 2009/05/15. doi: 10.1101/gr.086827.108 ; 19439512 10.1101/gr.086827.108 PMC2720184

[65] Postlethwait J, Amores A, Cresko W, Singer A, Yan YL. Subfunction partitioning, the teleost radiation and the annotation of the human genome. Trends in genetics: TIG. 2004;20(10):481–90. Epub 2004/09/15. doi: 10.1016/j.tig.2004.08.001 . 15363902 10.1016/j.tig.2004.08.001

[66] Aury JM, Jaillon O, Duret L, Noel B, Jubin C, Porcel BM, et al Global trends of whole-genome duplications revealed by the ciliate Paramecium tetraurelia. Nature. 2006;444(7116):171–8. Epub 2006/11/07. doi: 10.1038/nature05230 . 17086204 10.1038/nature05230

[67] Blanc G, Wolfe KH. Widespread paleopolyploidy in model plant species inferred from age distributions of duplicate genes. The Plant cell. 2004;16(7):1667–78. Epub 2004/06/23. doi: 10.1105/tpc.021345 ; 15208399 10.1105/tpc.021345 PMC514152

[68] Papp B, Pal C, Hurst LD. Dosage sensitivity and the evolution of gene families in yeast. Nature. 2003;424(6945):194–7. Epub 2003/07/11. doi: 10.1038/nature01771 . 12853957 10.1038/nature01771

[69] Hokamp K, McLysaght A, Wolfe KH. The 2R hypothesis and the human genome sequence. Journal of structural and functional genomics. 2003;3(1–4):95–110. Epub 2003/07/03. . 12836689

[70] Holland LZ. Chordate roots of the vertebrate nervous system: expanding the molecular toolkit. Nature reviews Neuroscience. 2009;10(10):736–46. Epub 2009/09/10. doi: 10.1038/nrn2703 . 19738625 10.1038/nrn2703

[71] Braasch I, Salzburger W, Meyer A. Asymmetric evolution in two fish-specifically duplicated receptor tyrosine kinase paralogons involved in teleost coloration. Molecular biology and evolution. 2006;23(6):1192–202. Epub 2006/03/21. doi: 10.1093/molbev/msk003 . 16547150 10.1093/molbev/msk003

[72] Brawand D, Wagner CE, Li YI, Malinsky M, Keller I, Fan S, et al The genomic substrate for adaptive radiation in African cichlid fish. Nature. 2014;513(7518):375–81. Epub 2014/09/05. doi: 10.1038/nature13726 . 25186727 10.1038/nature13726 PMC4353498

[73] Fares MA, Byrne KP, Wolfe KH. Rate asymmetry after genome duplication causes substantial long-branch attraction artifacts in the phylogeny of Saccharomyces species. Molecular biology and evolution. 2006;23(2):245–53. Epub 2005/10/07. doi: 10.1093/molbev/msj027 . 16207937 10.1093/molbev/msj027

[74] Steinke D, Salzburger W, Braasch I, Meyer A. Many genes in fish have species-specific asymmetric rates of molecular evolution. BMC genomics. 2006;7:20 Epub 2006/02/10. doi: 10.1186/1471-2164-7-20 ; 16466575 10.1186/1471-2164-7-20 PMC1413527

[75] Wagner GP, Takahashi K, Lynch V, Prohaska SJ, Fried C, Stadler PF, et al Molecular evolution of duplicated ray finned fish HoxA clusters: increased synonymous substitution rate and asymmetrical co-divergence of coding and non-coding sequences. Journal of molecular evolution. 2005;60(5):665–76. Epub 2005/06/29. doi: 10.1007/s00239-004-0252-z . 15983874 10.1007/s00239-004-0252-z

[76] Innan H, Kondrashov F. The evolution of gene duplications: classifying and distinguishing between models. Nature reviews Genetics. 2010;11(2):97–108. Epub 2010/01/07. doi: 10.1038/nrg2689 . 20051986 10.1038/nrg2689

[77] Raes J, Van de Peer Y. Gene duplication, the evolution of novel gene functions, and detecting functional divergence of duplicates in silico. Applied bioinformatics. 2003;2(2):91–101. Epub 2004/05/08. . 15130825

[78] Aparicio S, Chapman J, Stupka E, Putnam N, Chia JM, Dehal P, et al Whole-genome shotgun assembly and analysis of the genome of Fugu rubripes. Science. 2002;297(5585):1301–10. Epub 2002/07/27. doi: 10.1126/science.1072104 . 12142439 10.1126/science.1072104

[79] Venkatesh B, Kirkness EF, Loh YH, Halpern AL, Lee AP, Johnson J, et al Ancient noncoding elements conserved in the human genome. Science. 2006;314(5807):1892 Epub 2006/12/23. doi: 10.1126/science.1130708 . 17185593 10.1126/science.1130708

[80] Lee AP, Kerk SY, Tan YY, Brenner S, Venkatesh B. Ancient vertebrate conserved noncoding elements have been evolving rapidly in teleost fishes. Molecular biology and evolution. 2011;28(3):1205–15. Epub 2010/11/18. doi: 10.1093/molbev/msq304 . 21081479 10.1093/molbev/msq304

[81] Woolfe A, Elgar G. Organization of conserved elements near key developmental regulators in vertebrate genomes. Advances in genetics. 2008;61:307–38. Epub 2008/02/20. doi: 10.1016/S0065-2660(07)00012-0 . 18282512 10.1016/S0065-2660(07)00012-0

[82] Rytkonen KT, Prokkola JM, Salonen V, Nikinmaa M. Transcriptional divergence of the duplicated hypoxia-inducible factor alpha genes in zebrafish. Gene. 2014;541(1):60–6. Epub 2014/03/13. doi: 10.1016/j.gene.2014.03.007 . 24613281 10.1016/j.gene.2014.03.007

