# Supplementary figures and images for: Expansion by whole genome duplication and evolution of the sox gene family in teleost fish
Source: PLoS One. 2017 Jul 24;12(7):e0180936. doi: 10.1371/journal.pone.0180936 (PMC5524304; doi:10.1371/journal.pone.0180936)

SuppfigureS1

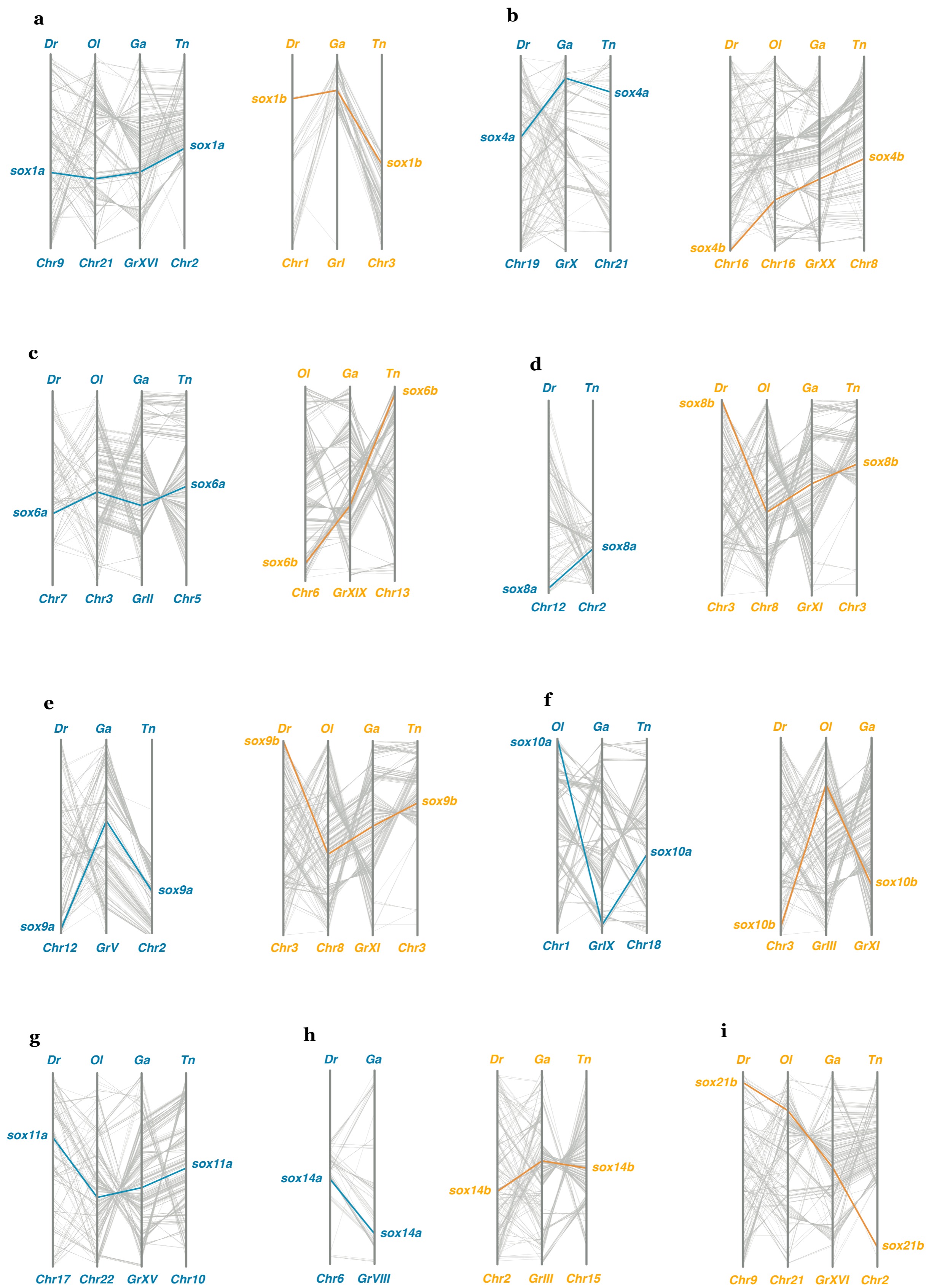

Supplement: S1 Fig — (a-i) Orthology relationships across four teleost species (D. rerio (Dr), O. latipes (Ol), G. aculeatus (Ga) and T. nigroviridis (Tn)) are represented. Soxa and soxb paralogs are respectively in blue and orange. (PDF) [file pone.0180936.s001.pdf]

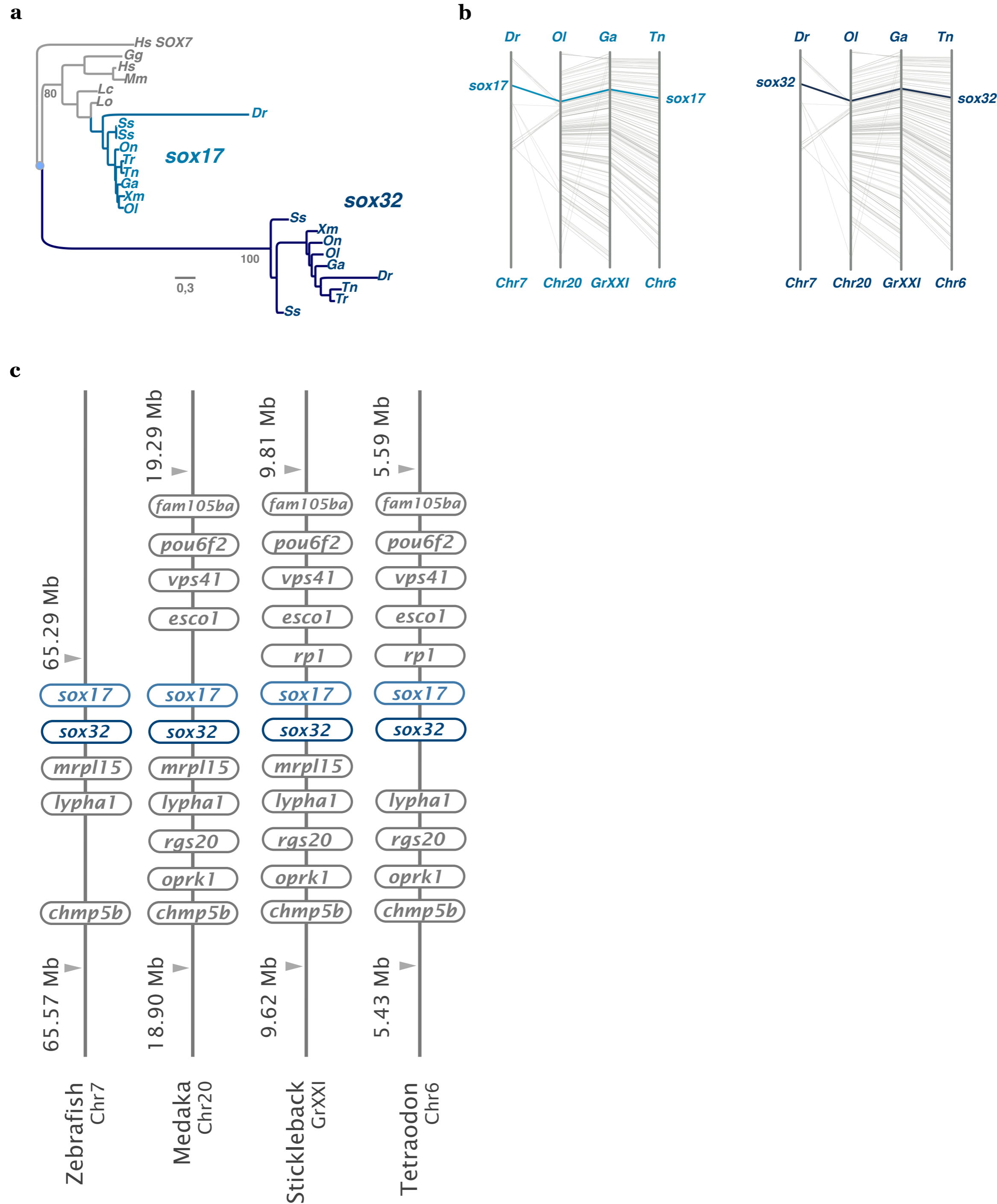

Supplement: S2 Fig — a) Phylogeny analysis of sox17/32. The phylogeny was computed using PhyML and based on protein sequences alignment (M. musculus (Mm), H. sapiens (Hs), G. gallus (Gg), L. chalumnae (Lc), L. oculatus (Lo), D. rerio (Dr), S. salar (Ss), O. niloticus (On), O. latipes (Ol), X. maculatus (Xm), T. nigroviridis (Tn), T. rubripes (Tr) and G. aculeatus (Ga)). Non-teleost sox17 orthologs are represented in grey. The tree is rooted with the human SOX7. b) Macrosynteny analysis of sox17/32. Orthology relationships across the four zebrafish D. rerio (Dr), medaka O. latipes (Ol), stickleback G. aculeatus (Ga) and Tetraodon T. nigroviridis (Tn) are represented. c) Microsynteny analysis of sox17/32. sox17/32-containing region in zebrafish, medaka, stickleback, and tetraodon genomes have been analyzed. Sox17 and sox32 are highlighted in light blue and dark blue respectively. (PDF) [file pone.0180936.s002.pdf]

SuppfigureS3

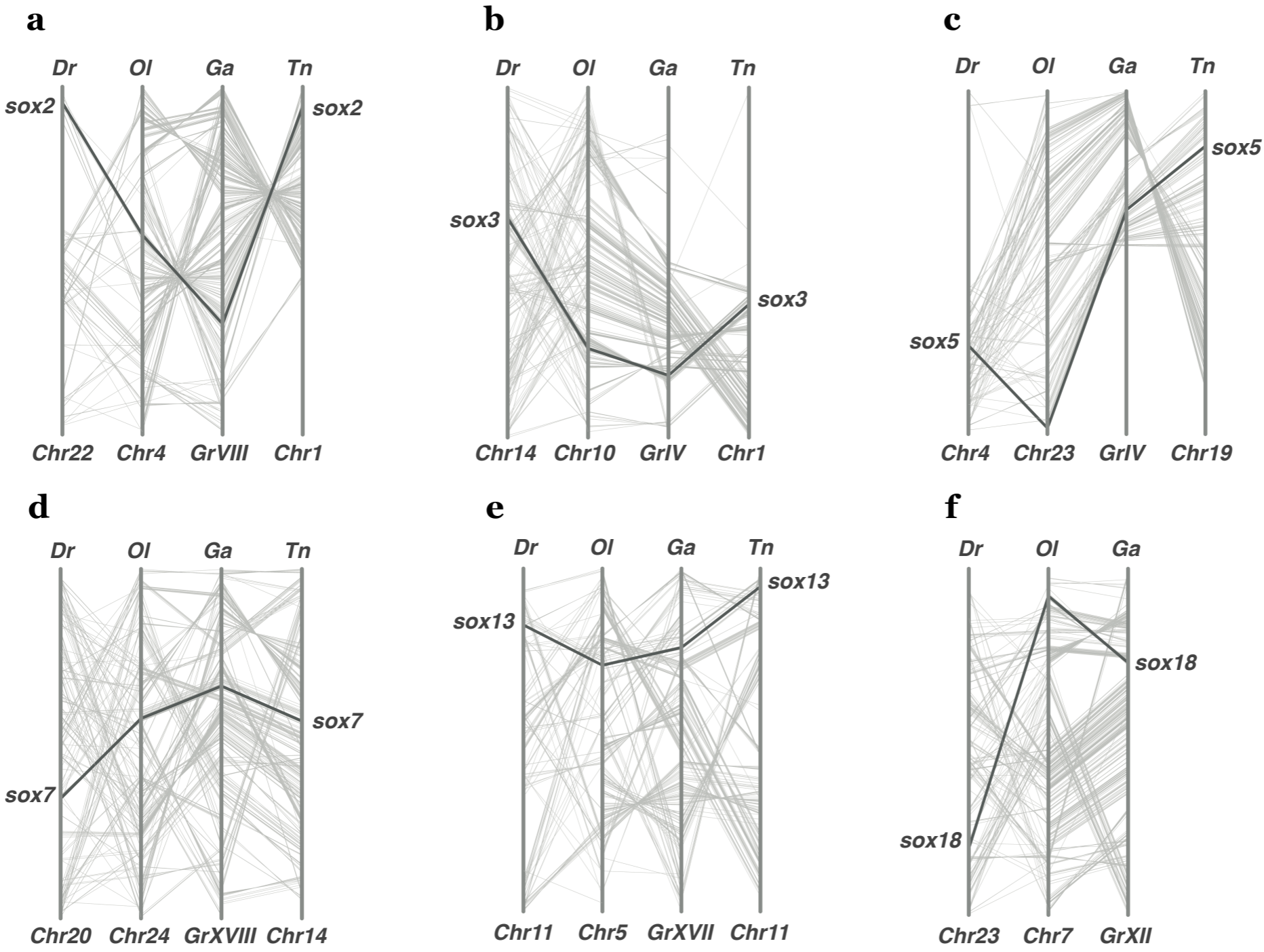

Supplement: S3 Fig — The four genomes of D. rerio (Dr), O. latipes (Ol), G. aculeatus (Ga) and T. nigroviridis (Tn) have been used. Grey lines connect orthologous genes on the different chromosomes (Chr) or linkage-group (Gr). The sox gene considered is highlighted in dark grey. a) sox2, b) sox3, c) sox5, d) sox7, e) sox13 and f) sox18. (PDF) [file pone.0180936.s003.pdf]
