# Supplementary material for: Expansion by whole genome duplication and evolution of the sox gene family in teleost fish
Source: PLoS One. 2017 Jul 24;12(7):e0180936. doi: 10.1371/journal.pone.0180936 (PMC5524304; doi:10.1371/journal.pone.0180936)
Supplement: S4 Fig — Log likelihood values (LnL) are obtained for each model (A, B or C). Parameters ωr, ωp, ωa and ωb are the dN/dS ratios for all branches, both paralogs branches, paralogs a branches and paralogs b branches, respectively. Tests were done by comparing twice the difference of likelihood values to a X2 distribution with degrees of freedom (df) equal to the difference in number of free parameters of each model. (PDF) [file pone.0180936.s004.pdf]

SuppFigureS4

	sox	Model	Ln*L* (np)	Estimates of ω ω^r	ω^p	ω^a	ω^b	Tests	df	p-value
B	1	A : One ratio	-6811.05	0.028				Model A *vs.* B	1	NS
		B : two ratio	-6809.73	0.022	0.030			Model B *vs.* C	1	<0.0001
		C : tree ratio	-6801.21	0.022		0.020	0.042			
	14	A : One ratio	-5843.90	0.036				Model A *vs.* B	1	<0.0001
		B : two ratio	-5829.83	0.017	0.049			Model B *vs.* C	1	<0.0001
		C : tree ratio	-5797.89	0.017		0.018	0.091			
	21	A : One ratio	-5821.282	0.02				Model A *vs.* B	1	NS
		B : two ratio	-5819.52	0.014	0.023			Model B *vs.* C	1	<0.0001
		C : tree ratio	-5811.101	0.013		0.073	0.021			
C	4	A : One ratio	-7271.80	0.032				Model A *vs.* B	1	NS
		B : two ratio	-7270.88	0.028	0.034			Model B *vs.* C	1	< 0.05
		C : tree ratio	-7268.92	0.029		0.040	0.029			
	11	A : One ratio	-8648.14	0.051				Model A *vs.* B	1	< 0.05
		B : two ratio	-8643.88	0.039	0.057			Model B *vs.* C	1	<0.0001
		C : tree ratio	-8631.77	0.039		0.038	0.077			
D	6	A : One ratio	-7304.04	0.047				Model A *vs.* B	1	< 0.05
		B : two ratio	-7301.12	0.036	0.053			Model B *vs.* C	1	<0.01
		C : tree ratio	-7295.66	0.036		0.040	0.068			
E	8	A : One ratio	-8608.62	0.044				Model A *vs.* B	1	<0.0001
		B : two ratio	-8594.53	0.021	0.056			Model B *vs.* C	1	<0.0001
		C : tree ratio	-8552.25	0.021		0.104	0.026			
	9	A : One ratio	-11100.46	0.029				Model A *vs.* B	1	<0.001
		B : two ratio	-11094.96	0.02	0.032			Model B *vs.* C	1	NS
		C : tree ratio	-11095.11	0.02		0.033	0.031			
	10	A : One ratio	-9970.81	0.051				Model A *vs.* B	1	< 0.05
		B : two ratio	-9968.2	0.04	0.056			Model B *vs.* C	1	< 0.05
		C : tree ratio	-9964.26	0.04		0.044	0.066			
