# Supplementary material for: Expansion by whole genome duplication and evolution of the sox gene family in teleost fish
Source: PLoS One. 2017 Jul 24;12(7):e0180936. doi: 10.1371/journal.pone.0180936 (PMC5524304; doi:10.1371/journal.pone.0180936)
Supplement: S5 Fig — We performed a student’s t-test based on the difference between means of the number of CNEs in the respective environment of the two paralogs. (PDF) [file pone.0180936.s005.pdf]

SuppfigureS5

	Sox4	Sox11	Sox8	Sox9	Sox10
Means of the number of CNEs for paralogue a	2.33	31.71	0.17	8.43	0
Means of the number of CNEs for paralogue b	6.86	3.00	5.00	25.00	3
t-test p-value	2,87.10-3	5.86.10-7	8,92.10-5	1,47.10-2	NA (constant data)
